# Sudden Onset, Rapidly Expansile, Cervical Cystic Hygroma in an Adult: A Rare Case with Unusual Presentation and Extensive Review of the Literature

**DOI:** 10.1155/2017/1061958

**Published:** 2017-05-24

**Authors:** Vivek Dokania, Anagha Rajguru, Harmanjot Kaur, Ketan Agarwal, Sujata Kanetkar, Prajakta Thakur, Femina Patel, Dhirajkumar Shukla

**Affiliations:** ^1^Department of ENT, Krishna Institute of Medical Sciences Deemed University, Karad 415110, India; ^2^Wyckoff Heights Medical Center, Brooklyn, NY 11237, USA; ^3^Department of Pathology, Krishna Institute of Medical Sciences Deemed University, Karad 415110, India; ^4^Dr. Vasantrao Pawar Medical College, Hospital & Research Center, Nashik 422003, India; ^5^Krishna Institute of Medical Sciences Deemed University, Karad 415110, India

## Abstract

Cystic hygroma (CH) is a benign infiltrative malformation of the lymphatic channels. We report a case of a 28-year-old Indian female who presented with rapidly enlarging right sided neck swelling over the posterior triangle since 5 days. Complete resection of CH is sometimes not amenable because of its infiltrative nature and involvement of surrounding vital structures. However, in our patient successful complete surgical resection was undertaken. The MRI findings of our patient were consistent with brachial cleft cyst; this posed a challenge in the diagnosis of CH. The histopathological analysis of the resected mass confirmed CH. CH is rare in adults and such an acute presentation is exceptionally atypical. History of prior trauma and infection are known etiological factors for adult CH; these were conspicuously absent in our patient. CH should be considered in the differentials of rapidly enlarging cystic swelling of posterior region of neck in adults. Optimal and timely management is necessary to achieve a favorable prognosis. Therefore, we report a case of rapidly enlarging cervical CH in an adult along with extensive literature review to have a better understanding regarding epidemiology, etiopathogenesis, clinical presentation, optimal management, and prognosis of such a rare entity in adults.

## 1. Introduction

Cystic hygroma is a subtype of lymphangioma and is most commonly a congenital lesion [1]. It is synonymous with cystic lymphangioma and macrocystic lymphatic malformation. It is an aberrant proliferation of lymphatic vessels resulting from abnormal development of lymphatic system. Histologically, it exhibits large macroscopic cystic spaces.

First case of lymphatic malformation (LM) was described by Redenbacher in 1828 [2]. Wernher [3] coined the term “hygroma” and later described a histological classification system for LMs into 3 types: cystic, capillary, and cavernous. Sabin [4] in 1990 and later Goetsch [5] in 1938 proposed the theory of centrifugal lymphatic system development. Sabin described the embryological derivation of lymphatic tissues as arising from 5 primitive lymphatic buds and postulated that abnormal sequestration of these buds gives rise to lymphatic malformations. Other theories, such as the centripetal theory of lymphatic development, have also been proposed [6].

CH can affect any anatomic subsites in human body [7–9]; however, most of these lesions are seen in head and neck region (~75), probably because of rich lymphatics in this area. CH usually affects children under 2 years of age (80–90%) with incidence of 1.2 to 2.8 per 100,000 infants [10, 11]. CH does not show any gender preference; both genders are equally affected [12, 13].

CH presenting in adulthood is rarely seen [14, 15]. The etiology for CH arising in adults is controversial but it is thought to arise due to acquired process like infection, trauma (including surgery), or lymphatic obstruction [12, 16, 17].

To date there are only few cases of adult cervical CH described in literature and the optimum management is still challenging. Preoperative diagnosis is difficult in adults and often patients are misdiagnosed. Patients with CH are frequently misdiagnosed as brachial cleft cysts on imaging as in the case reported here. Definitive diagnosis can be reached only after histopathological examination of the surgical specimen.

Complete surgical removal is an important treatment modality [18]; however, the lesion tends to spread along vital structure; therefore, in such patients inductive complete surgical removal cannot be achieved. Several adjunctive therapies have been shown to be beneficial in recurrent or inaccessible lesion but these are not used commonly. Sclerosis by chemical drug [19–22] is another effective treatment option.

Just as with brachial cleft cyst and thyroglossal duct cyst, it is important to consider LM in one's differential diagnosis in the setting of an adult patient with a cystic mass of neck. For this reason, we describe a case of adult cervical cystic hygroma along with literature discussion for better understanding of disease process in adult and optimal management options.

## 2. Case Presentation

In our case, a 28-year-old Indian female presented with painless right side neck swelling since 5 days. She had no compressive neck symptoms like difficulty swallowing or difficulty breathing. There was no history of preceding neck trauma or upper respiratory tract infection.

Physical examination showed a solitary swelling on the right, posterior aspect of neck measuring about 6.5 × 3 cm. Margins were ill-defined. The surface on palpation was lobular, soft in consistency, and freely movable. The swelling was immobile with deglutition. Overlying skin illustrated no erythema, warmth, or tenderness. No cervical lymphadenopathy was noted.

**Figure 1:**
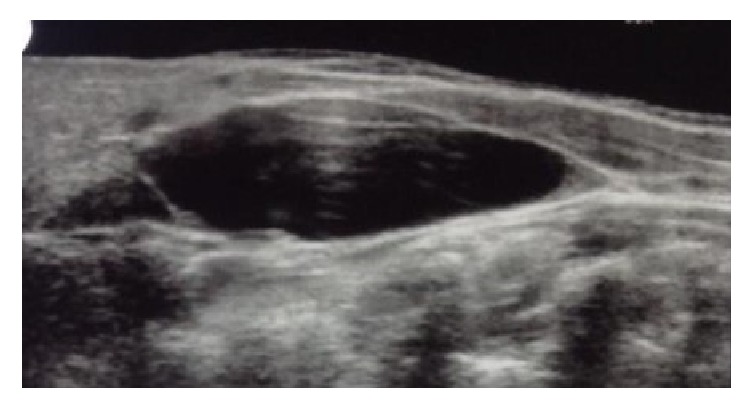
USG showing cystic neck lesion.

Ultrasound scanning showed a large elliptical 8 × 7 × 3 cm cystic lesion posteromedial to right sternocleidomastoid muscle extending to right submandibular gland medially. Deep wall was abutting carotid artery and jugular vein, pushing them posteriorly. Cyst shows peripheral thin septae and tiny loculations. Cyst wall and interlocular septae were avascular (Figure 1). On the basis of the above-mentioned USG findings, a diagnosis favoring cystic hygroma was reported by the radiologist and MRI was planned next so as to get a more accurate radiological diagnosis.

**Figure 2:**
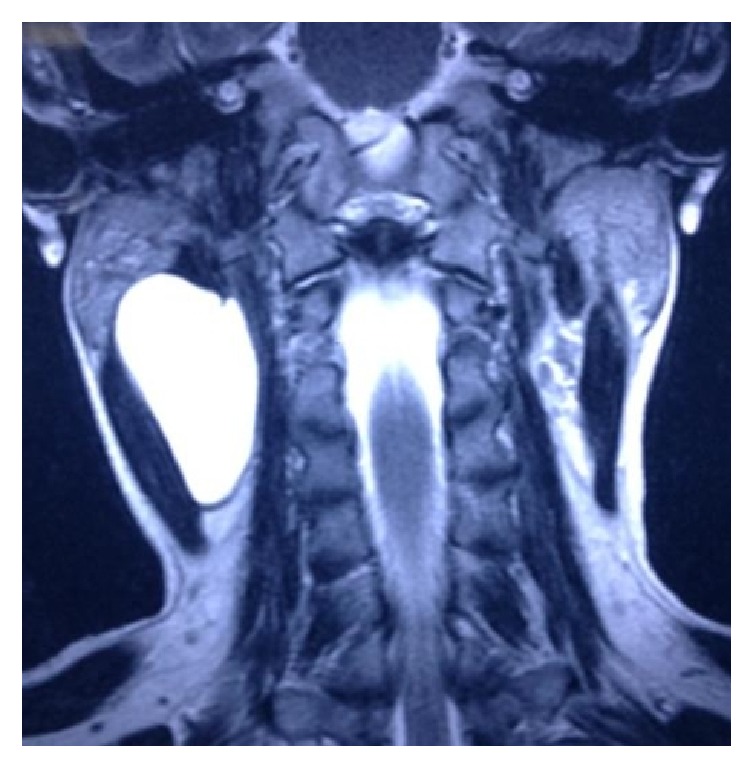
T2 weighted image in coronal view revealing a macrocystic lesion in the right side posterior cervical triangle.

**Figure 3:**
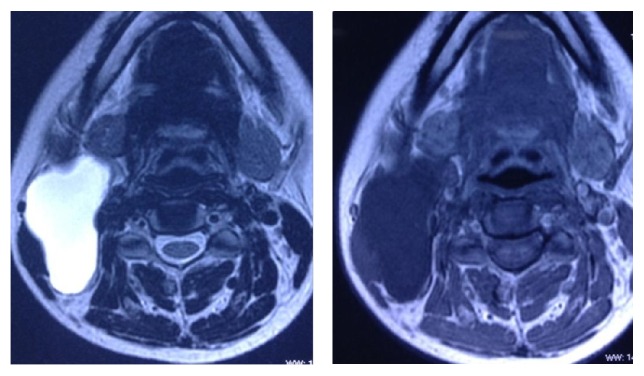
T2 and T1 weighted images in axial view revealing a macrocystic lesion in the right side posterior cervical triangle.

Magnetic resonance imaging findings showed a thin walled multilobular cystic mass with a size of 66.0 × 53.7 × 27.0 mm (CC × AP × Trans) in intermuscular plane on right side of neck. Inferiorly it extended up to the level of vocal cord; laterally it abutted the sternocleidomastoid muscle and parotid gland. Medially it abutted the carotid sheath and scalene muscle and posteriorly it abutted the submandibular gland. No communication to pharynx is seen and no inflammatory changes were noted around it. The mass appeared hyperintense on T2-weighted images and hypointense on T1-weighted images (Figures 2 and 3). This is a stage III lesion according to the staging system proposed by De Serres. In our patient the radiologist reported that the lesion could also be a second branchial cleft cyst.

Because of contrary between USG and MRI findings, FNA was conducted to get a probable diagnosis. Yellow viscous fluid was aspirated from the cyst and on cytological analysis was found to contain numerous lymphocytes, making cystic hygroma a probable diagnosis.

**Figure 4:**
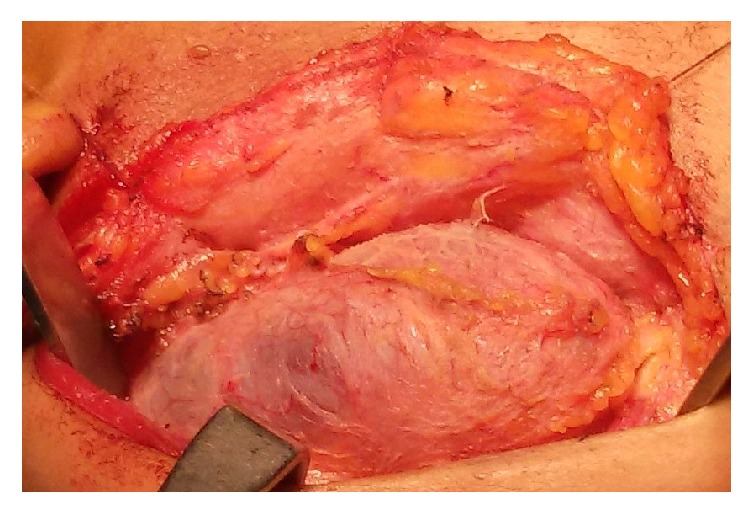
Intraoperative photograph of large cystic lesion dissected from right side of neck.

**Figure 5:**
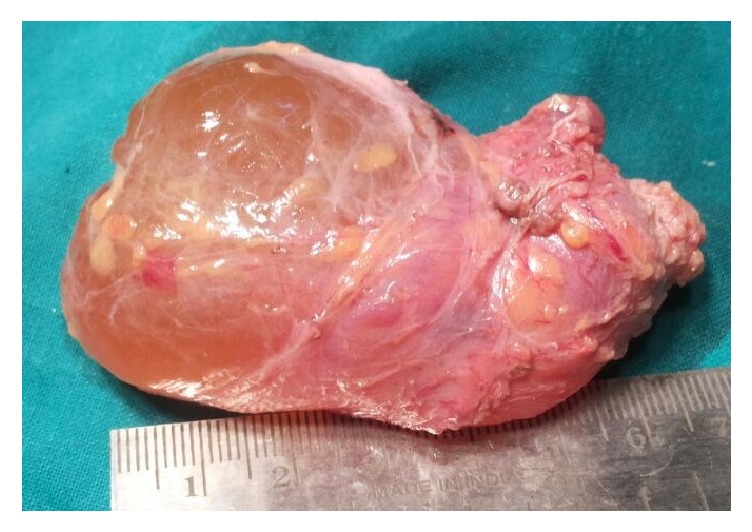
Surgical specimen.

Surgery was conducted under general anaesthesia. A horizontal incision was given over the maximum bulge of the underlying cyst and the overlying skin; subcutaneous fat with platysmal flap was raised apart exposing the cystic mass which measured approximately 7 cm along the widest diameter (Figure 4). Cyst was dissected in planes and noted to be lying over internal jugular vein, carotid artery, and spinal accessory nerve. The cyst was pushing sternocleidomastoid muscle laterally, extending up to parotid gland superiorly. Cyst was excised (Figure 5) and surgical drain was kept fixed. Suturing was done in two layers and dressing was applied. Drain was removed on 3rd postoperative day and dressing with sutures was removed on 7th postoperative day, displaying a healthy wound. No recurrence or complication has been noted until one-year postoperative follow-up.

**Figure 6:**
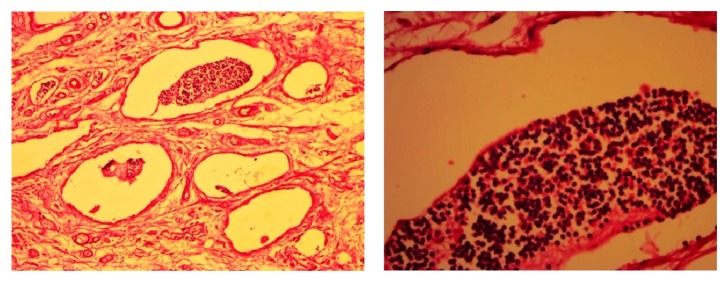
Hematoxylin and eosin stained low (100x) and high (400x) resolution histological images showing endothelium lined cystic spaces containing lymphocytes.

Histopathological findings revealed cyst wall showing endothelium lined channels and collections of lymphoid tissue confirming a diagnosis of cystic hygroma (Figure 6).

## 3. Discussion

Lymphatic malformations are uncommon benign, congenital malformations of the lymphatic system that involve the skin and subcutaneous tissues.

Several staging and classification system have been opted for better diagnosis and management. Smith et al. [23] described lymphatic malformation as microcystic, macrocystic, or mixed variant, with macrocystic containing cysts more than 2 cm in diameter. Cystic hygroma is a misnomer for macrocystic lymphatic malformation. Kennedy [12] classified these lesions into 4 types: superficial cutaneous, cavernous, cystic hygroma, and diffuse systematic. Jackson [24] classified vascular malformations into 2 types: low flow and high flow. Lymphatic malformations were classified as low flow lesions. de Serres [25] proposed a staging system according to location and extent of lesions: stage 1 is unilateral infrahyoid, stage 2 is unilateral suprahyoid, stage 3 is unilateral infrahyoid and suprahyoid, stage 4 is bilateral infrahyoid, and stage 5 is bilateral infrahyoid and suprahyoid.

75–80% of CHs are located in head and neck region [26–28]. In the neck, they are typically located in posterior triangle [12, 29–33]. Rarely, it is seen in adult in their 4th to 6th decades and its occurrence in younger adult, as in our patient, is even less common [12, 28, 29, 34]. Fewer than 150 cases of adult LMs have been reported in literature [11, 35, 36].

Schefter et al. [37] reported the largest series of adult CH, with 32 patients. Naidu and McCalla [38] did an extensive review of literature on lymphatic malformations on 91 adult patients and, in addition, reported the oldest case of cystic hygroma in a 91-year-old woman. The ages of 89 of the 91 patients were provided, with the average age at presentation of 36.6 years and ranging from 16 to 86 years. 45% of these patients were male. Location wise, no significant predilection was shown in their study (46.4% left, 50% right). However, Schefter et al. noted right side dominance.

Cystic hygroma varies from 1.0 to 30.0 cm in size; the mean size in Stromberg's series was 8.0 cm [19].

There are several genetic syndromes associated with cystic hygromas, although most commonly seen in Turner syndrome, associated with other chromosomal abnormalities such as trisomies 13, 18, and 21 and with Noonan syndrome. Isolated cystic hygromas can be inherited as an autosomal recessive pattern for which parents are “silent” carriers [39–43].

Some authors attribute adult LMs to delayed proliferation of congenital lymphoid rests. Acquired processes like infection, trauma (including surgery), or lymphatic obstruction have also been linked to adult LMs. Aneeskumar et al. [44] suggested that trauma could trigger formation of lymphangioma. Mhoon et al. [45] reported a scrotal swelling after trauma.

Clinical presentation depends on the location, size, and rate of growth of malformation. Extensive lesions of the floor of mouth, oropharynx, or neck result in airway insufficiency. Cervical lesions can lead to compressive lesions like dysphagia when compressing esophagus or dyspnea on tracheal or laryngeal compression. Gorham syndrome [46] or vanishing bone syndrome is a type of lymphatic malformation that involves bone and surrounding tissue. Hypertrophy of bones and soft tissues often leads to macroglossia, macrocheilia, and macrotia. Zheng et al. [47] found that 83% of LMs patients were associated with bone deformity and 33% with bone morphogenetic abnormalities. Lymphopenia can sometimes also be an associated feature [48].

Various diagnostic modalities have been mentioned in literature and imaging methods like USG, MRI, and CT scan have been used prior to treatment; however, imaging preferences vary by cost, convenience, and resolution. Preoperative imaging is even important to look for intrathoracic extension which is present in 10% of cases [49]. FNAC is recommended by few authors but some consider it as a source of inflicting infection and increasing risk of bleeding and recurrence [36, 50]. Usually, a yellow fluid with mature lymphocytes and histiocytes are aspirated. USG is mostly the first-order study of choice to investigate a suspected mass of neck because of its noninvasiveness, low cost, and nonuse of ionizing radiation [51]. USG shows fluid filled cystic cavities with or without loculation. On CT, the lesion is seen as homogeneous cystic masses with no enhancement after intravenous contrast injection [52]. On MRI, the lesion has low to intermediate intensity on T1- weighted image, hyperintensity on T2-weighted image with no enhancement after contrast injection [52, 53]. Sometimes LMs with internal hemorrhage display low signal intensity on T2 weighted image.

Many treatment methods have been documented in the literature, including surgery, various sclerotherapy, radiotherapy, and laser therapy. Kennedy et al. [54] recommended awaiting spontaneous resolution in order to avoid potential hazards of surgery. Spontaneous regression is rarely seen and only 1.6–16.0% has been reported [47, 55, 56]. Surgery used to be the mainstay of treatment and remains the treatment of choice for LMs. Their proclivity to infiltrate nearby neurovascular structure and muscles makes surgical excision difficult, especially in children [29, 44, 54, 57–59]. In adults CH is well circumscribed, hence making complete surgical excision relatively easier compared to pediatric population [60]. Wiggs and Sismanis [14] recommended earliest surgical excision of adult CH so as to avoid prospective difficult excision following infection. Formal neck dissection removing abnormal lymphatic tissues, sometimes including a parotidectomy, has also been recommended by some authors [50]. Complications of resection include infection, bleeding, hematoma, and postoperative seromas. Injury to facial, hypoglossal, glossopharyngeal, recurrent laryngeal, and lingual nerves has been reported [34, 39, 54]. Radiotherapy, sclerotherapy, electrocoagulation, cryotherapy, and laser therapy have been recommended as primary or adjunctive treatment for extensive and diffuse malformation. Radiotherapy is effective in inhibiting microcystic lesions but has been abandoned due to the risk of malignant transformation [61]. Various sclerosing agents have been used including ethanol, doxycycline, quinine, hypertonic glucose, corticosteroids, sodium morrhuate, bleomycin, and OK-432; bleomycin and OK-432 remain the mainstay therapeutic agent for LMs. OK-432 (picibanil) is obtained from a lyophilized mixture of Group A pyogenes streptococcus and because of immunomodulating effect it increases several cytokines, such as interleukin- (IL-) 1, IL-2, interferon gamma, IL-6, and tumour necrosis factor, thus increasing endothelial permeability, accelerating lymphatic drainage, and fostering retraction of cystic cavity [62, 63]. Yura et al. [64] and Ogita et al. [65] first reported use of intralesional bleomycin and OK-432 with excellent results. Complete resolution was noted in eight out of nine patients treated with intralesional injection of OK-432 by Ogita et al. Sainsbury et al. [66] observed 100% overall response rate and 40% complete response rate in patients treated with intralesional bleomycin. Burrows et al. [67] observed a response rate of about 83% in patients with LMs treated with intralesional doxycycline. There are concerns regarding adverse reactions of sclerosing agents. Bleomycin increases risk of pulmonary fibrosis, OK-432 causes sepsis, shock, and myalgia, and doxycycline causes neural damage [68]. Laser therapy is effective for superficial microcystic lesions. CO2 laser, Nd:YAG laser, pulsed dye laser, and diode lasers are available options [69]. Zhou et al. [70] described a treatment decision algorithm for head and neck LMs.

Recurrence of cystic hygroma after excision is influenced by various factors like location of lesion relative to hyoid bone, presence/absence of encapsulation, and partial versus complete excision. Ricciardelli and Richardson [34] reported that recurrence is related to the location of lesion relative to hyoid bone, with lesions located above the hyoid having higher chances of recurrence. Fliegelman et al. [20] reported that recurrence is related to histological encapsulation of lesion. Recurrence rate of 10–15% has been reported with partial surgical excision. Most common lesions of posterior triangle of neck are mostly solids and include inflammatory and metastatic adenopathies, lymphomas, lipomas, and neurofibromatosis [71, 72]. When evaluating cystic lesions of neck, the most common differentials are thyroglossal cysts, followed by brachial cleft cysts and lymphangiomas [73, 74].

## Abbreviations

CH: Cystic hygroma
LM: Lymphatic malformation
LMs: Lymphatic malformations
CT: Computed tomography
MRI: Magnetic resonance imaging
USG: Ultrasound imaging
FNA: Fine needle aspiration
FNAC: Fine needle aspiration cytology.

## References

[1] Chappius I. I. P. (1995). Current aspects of cystic lymphangioma in the neck. *Archives de Pédiatrie*.

[2] Redenbacher E. A. H. (1828). *De Ranula Sublingual, Speciali, Cum Casa Congenital*.

[3] Wernher A., Heyer G. F. (1843). Die angeborenen Zysten-Hygrome und die ihnen verwandten geschwülste. *Anatomischer, Diagnostischer Und Therapeutischer Beziehung*.

[4] Sabin F. R. (1909). The lymphatic system in human embryos, with a consideration of the morphology of the system as a whole. *American Journal of Anatomy*.

[5] Goetsch E. (1938). Hygroma colli cysticum and hygroma axillae. *Archives of Surgery*.

[6] Huntington G. S. (1911). *The Anatomy and Development of the Systematic Lymphatic Vessels in the Domestic Cat*.

[7] Rodriguez P. M., Navarro J. R. (2009). Cystic hygroma. *An Orl Mex*.

[8] Robledo-Ogazón F., Vargas-Rivas A. E., Alvarado-Aparicio A. (2004). Adrenal gland lymphangiomas. A case report. *Cirugia y Cirujanos*.

[9] Barreto Z. R., Gracia L. J., Tanimoto M. A. (2006). Jejunal lymphangioma. *Revista de Gastroenterología de México*.

[10] Guruprasad Y., Chauhan D. S. (2012). Cervical cystic hygroma. *Journal of Maxillofacial and Oral Surgery*.

[11] Gow L., Gulati R., Khan A., Mihaimeed F. (2011). Adult-onset cystic hygroma: a case report and review of management. *Grand Rounds*.

[12] Kennedy T. L. (1989). Cystic hygroma-lymphangioma: a rare and still unclear entity. *Laryngoscope*.

[13] Hancock B. J., St-Vil D., Luks F. I., Di Lorenzo M., Blanchard H. (1992). Complications of lymphangiomas in children. *Journal of Pediatric Surgery*.

[14] Wiggs W. J., Sismanis A. (1994). Cystic hydroma in the adult: two case reports. *Otolaryngology—Head and Neck Surgery*.

[15] Obon P. J. (1993). Reported case of cystic lymphangioma in an adult. *Acta Otorrinolaringologica Española*.

[16] Nosan D. K., Martin D. S., Stith J. A. (1995). Lymphangioma presenting as a delayed posttraumatic expanding neck mass. *American Journal of Otolaryngology—Head and Neck Medicine and Surgery*.

[17] Gleason T. J., Yuh W. T. C., Harris K. G., Tali E. T., Mueller D. P. (1993). Traumatic cervical cystic lymphangioma in an adult. *Annals of Otology, Rhinology & Laryngology*.

[18] Farmand M., Kuttenberger J. J. (1996). A new therapeutic concept for the treatment of cystic hygroma. *Oral Surgery, Oral Medicine, Oral Pathology, Oral Radiology, and Endodontics*.

[19] Stromberg B. V., Weeks P. M., Jr. Wray R. C. (1976). Treatment of cystic hygroma. *Southern Medical Journal*.

[20] Fliegelman L. J., Friedland D., Brandwein M., Rothschild M. (2000). Lymphatic malformation: predictive factors for recurrence. *Otolaryngology—Head and Neck Surgery*.

[21] Chong K. T., Ong C. L. (1997). Cystic hygroma in adulthood. *Singapore Medical Journal*.

[22] Mikhail M., Kennedy R., Cramer B., Smith T. (1995). Sclerosing of recurrent lymphangioma using OK-432. *Journal of Pediatric Surgery*.

[23] Smith R. J., Burke D. K., Sato Y., Poust R. I., Kimura K., Bauman N. M. (1996). OK-432 therapy for lymphangiomas. *Archives of Otolaryngology—Head and Neck Surgery*.

[24] Jackson I. T., Carreho R., Potparic Z., Hussain K. (1993). Hemangiomas, vascular malformations, and lymphovenous malformations: classification and methods of treatment. *Plastic and Reconstructive Surgery*.

[25] de Serres L. M., Sie K. C. Y., Richardson M. A. (1995). Lymphatic malformations of the head and neck. A proposal for staging. *Archives of Otolaryngology—Head and Neck Surgery*.

[26] Godin D. A., Guarisco J. L. (1997). Cystic hygromas of the head and neck. J La State Med Soc. *Guarisco JL. Cystic hygromas of the head and neck. J La State Med Soc*.

[27] Mahboubi S., Potsic W. P. (1989). Computed tomography of cervical cystic hygroma in the neck. *International Journal of Pediatric Otorhinolaryngology*.

[28] Sichel J.-Y., Udassin R., Gozal D., Koplewitz B. Z., Dano I., Eliashar R. (2004). OK-432 therapy for cervical lymphangioma. *Laryngoscope*.

[29] Baer S., Davis J. (1989). Cystic hygroma presenting in adulthood. *The Journal of Laryngology & Otology*.

[30] Guarisco J. L. (1991). Congenital head and neck masses in infants and children. Part II. *Ear, Nose and Throat Journal*.

[31] Çelenk F., Ceylan A., Köybaşıoğlu A., Gönül İ. I. (2006). Erişkin hastada servikal kistik lenfanjiom: olgu sunumu. *KBB-Forum*.

[32] Güner A., Aydın A., Çelik F. (2006). Cystic hygromas in adults: reports of two cases. *Bakırköy Tıp Dergisi*.

[33] Gidvani V. K., Bhowmick S. K. (1999). Midline posterior cervical cystic hygroma. *Southern Medical Journal*.

[34] Ricciardelli E. J., Richardson M. A. (1991). Cervicofacial cystic hygroma: patterns of recurrence and management of the difficult case. *Archives of Otolaryngology—Head and Neck Surgery*.

[35] Sherman B. E., Kendall K. (2001). A unique case of the rapid onset of a large cystic hygroma in the adult. *American Journal of Otolaryngology—Head and Neck Medicine and Surgery*.

[36] Suk S., Sheridan M., Saenger J. S. (1997). Adult lymphangioma: a case report. *Ear, Nose and Throat Journal*.

[37] Schefter R. P., Olsen K. D., Gaffey T. A. (1985). Cervical lymphangioma in the adult. *Otolaryngology—Head and Neck Surgery*.

[38] Naidu S. I., McCalla M. R. (2004). Lymphatic Malformations of the Head and Neck in Adults: A Case Report and Review of the Literature. *Annals of Otology, Rhinology & Laryngology*.

[39] Chen C.-P., Chern S.-R., Chang C.-L. (2000). Prenatal diagnosis and genetic analysis of X chromosome polysomy 49,XXXXY. *Prenatal Diagnosis*.

[40] Parker G., Harnsberger H., Smoker W. (1991). The anterior and posterior cervical spaces. *Seminars in Ultrasound, CT and MRI*.

[41] Bloom D. C., Perkins J. A., Manning S. C. (2004). Management of lymphatic malformations. *Current Opinion in Otolaryngology and Head and Neck Surgery*.

[42] Carta G., Iovenitti P., D'Alfonso A., Mascaretti G., Moscarini M. (1999). Fetal malformations and chromosome abnormalities diagnosed at the center of prenatal diagnosis of the university of aquila in the 1995–1998 triennium. *Minerva Ginecologica*.

[43] Gallagher P. G., Mahoney M. J., Gosche J. R. (1999). Cystic hygroma in the fetus and newborn. *Seminars in Perinatology*.

[44] Aneeshkumar M. K., Kale S., Kabbani M., David V. C. (2005). Cystic lymphangioma in adults: can trauma be the trigger?. *European Archives of Oto-Rhino-Laryngology*.

[45] Mhoon J. M., Redman J. F., Siebert J. J. (2002). Scrotal enlargement in boys with a history of scrotal trauma: two unusual findings. *Southern Medical Journal*.

[46] Radhakrishnan K., Rockson S. G. (2008). Gorham's disease: an osseous disease of lymphangiogenesis?. *Annals of the New York Academy of Sciences*.

[47] Zheng J. W., Zhou Q., Yang X. J. (2010). Treatment guideline for hemangiomas and vascular malformations of the head and neck. *Head & Neck*.

[48] Perkins J. A., Tempero R. M., Hannibal M. C., Manning S. C. (2007). Clinical outcomes in lymphocytopenic lymphatic malformation patients. *Lymphatic Research and Biology*.

[49] Morley S. E., Ramesar K. C. R. B., Macleod D. A. D. (1999). Cystic hygroma in an adult: a case report. *Journal of the Royal College of Surgeons of Edinburgh*.

[50] Curran A. J., Malik N., McShane D., Timon C. V. (1996). Surgical management of lymphangiomas in adults. *The Journal of Laryngology & Otology*.

[51] Kumar S., Kumar S., Kumar S., Prakash V., Kumar V. (2014). Dumbbell-shaped lymphangioma of neck and thorax. *National Journal of Maxillofacial Surgery*.

[52] Romeo V., Maurea S., Mainenti P. P. (2015). Correlative imaging of cystic lymphangiomas: ultrasound, CT and MRI comparison. *Acta Radiologica Open*.

[53] Arnold R., Chaudry G. (2011). Diagnostic imaging of vascular anomalies. *Clinics in Plastic Surgery*.

[54] Kennedy T. L., Whitaker M., Pellitteri P., Wood W. E. (2001). Cystic hygroma/lymphangioma: a rational approach to management. *Laryngoscope*.

[55] Boyd J. B., Mulliken J. B., Kaban L. B., Upton J., Murray J. E. (1984). Skeletal changes associated with vascular malformations. *Plastic and Reconstructive Surgery*.

[56] Gross R. E., Gross R. E. (1953). Cystic hydroma. *The Surgery of Infancy And Childhood*.

[57] Antoniades K., Kiziridou A., Psimopoulou M. (2000). Traumatic cervical cystic hygroma. *International Journal of Oral and Maxillofacial Surgery*.

[58] Karkos P. D., Spencer M. G., Lee M., Hamid B. N. (2005). Cervical cystic hygroma/lymphangioma: an acquired idiopathic late presentation. *Journal of Laryngology and Otology*.

[59] Oakes M. J., Sherman B. E. (2004). Cystic hygroma in a tactical aviator: a case report. *Military Medicine*.

[60] Nussbaum M., Buchwald R. P. (1981). Adult cystic hygroma. *American Journal of Otolaryngology—Head and Neck Medicine and Surgery*.

[61] Di Carlo I., Gayet B. (1996). Lymphangioma of the diaphragm (first case report). *Surgery Today*.

[62] Sung M.-W., Lee D.-W., Kim D.-Y. (2001). Sclerotherapy with picibanil (OK-432) for congenital lymphatic malformation in the head and neck. *Laryngoscope*.

[63] Ogita S., Tsuto T., Nakamura K., Deguchi E., Tokiwa K., Iwai N. (1996). OK-432 therapy for lymphangioma in children: why and how does it work?. *Journal of Pediatric Surgery*.

[64] Yura J., Hashimoto T., Tsuruga N., Shibata K. (1977). Bleomycin treatment for cystic hygroma in children. *Nihon Geka Hokan*.

[65] Ogita S., Tsuto T., Tokiwa T., Takahashi T. (1987). Treatment of cystic hygroma in children with special reference to OK-432 therapy. *Zeitschrift fur Kinderchirurgie*.

[66] Sainsbury D. C. G., Kessell G., Fall A. J., Hampton F. J., Guhan A., Muir T. (2011). Intralesional bleomycin injection treatment for vascular birthmarks: a 5-year experience at a single United Kingdom unit. *Plastic and Reconstructive Surgery*.

[67] Burrows P. E., Mitri R. K., Alomari A. (2008). Percutaneous sclerotherapy of lymphatic malformations with doxycycline. *Lymphatic Research and Biology*.

[68] Buckmiller L. M., Richter G. T., Suen J. Y. (2010). Diagnosis and management of hemangiomas and vascular malformations of the head and neck. *Oral Diseases*.

[69] Angiero F., Benedicenti S., Benedicenti A., Arcieri K., Bernè E. (2009). Head and neck hemangiomas in pediatric patients treated with endolesional 980 nm diode laser. *Photomedicine and Laser Surgery*.

[70] Zhou Q., Zheng J. W., Mai H. M. (2011). Treatment guidelines of lymphatic malformations of the head and neck. *Oral Oncology*.

[71] Parker G. D., Harsnberger H. R. (1991). Radiology evaluation of the normal and diseased posterior cervical space. *American Journal of Roentgenology*.

[72] Parker G. D., Harsnberger H. R., Smoker W. R. K. (1991). The anterior and posterior cervical spaces. *Seminars in Ultrasound, CT and MRI*.

[73] Koeller K. K., Alamo L., Adair C. F., Smirniotopoulos J. G. (1999). From the archives of the AFIP. Congenital cystic masses of the neck: radiologic-pathologic correlation. *Radiographics*.

[74] Brea-Álvarez B., Roldán-Fidalgo A. (2015). Cysts in the posterior triangle of the neck in adults. *Acta Otorrinolaringologica*.

